# Patients’ evaluation of aftercare following hospitalization for COVID-19: satisfaction and unmet needs

**DOI:** 10.1186/s12931-024-02748-8

**Published:** 2024-03-29

**Authors:** Julia C. Berentschot, Willemijn A. de Ridder, L. Martine Bek, Majanka H. Heijenbrok-Kal, Gert-Jan Braunstahl, Sylvia C. Remerie, Yvonne Stuip, Gerard M. Ribbers, Joachim G. J. V. Aerts, Erwin Ista, Merel E. Hellemons, Rita J. G. van den Berg-Emons, the CO-FLOW Collaboration Group, Sieshem Bindraban, Wouter J. B. Blox, Jasper van Bommel, Shai A. Gajadin, Michel E. van Genderen, Diederik A. M. P. J. Gommers, Stephanie van Loon-Kooij, Rutger Osterthun, Laurien Oswald, Ronald N. van Rossem, Herbert J. van de Sande, Robert van der Stoep, Janette J. Tazmi-Staal, Chantal J. C. Luijkx, Markus P. J. M. Wijffels, Eva G. Willems

**Affiliations:** 1 Department of Respiratory Medicine, Erasmus MC, University Medical Center Rotterdam, Dr. Molewaterplein 40, 3015 GD Rotterdam, The Netherlands; 2 Department of Rehabilitation Medicine, Erasmus MC, University Medical Center Rotterdam, Rotterdam, The Netherlands; 3 Department of Plastic, Reconstructive and Hand Surgery, Erasmus MC, University Medical Center Rotterdam, Rotterdam, The Netherlands; 4 Hand and Wrist Center, Xpert Clinics, Eindhoven, The Netherlands; 5 Center for Hand Therapy, Xpert Handtherapie, Eindhoven, The Netherlands; 6 Rijndam Rehabilitation, Rotterdam, the Netherlands; 7 Department of Respiratory Medicine, Franciscus Gasthuis, Rotterdam, The Netherlands; 8 Zorghoek Bergschenhoek, Bergschenhoek, The Netherlands; 9 Department of Internal Medicine, Nursing Science, Erasmus MC, University Medical Center Rotterdam, Rotterdam, The Netherlands; 10 Departments of Neonatal and Pediatric Intensive Care, Division of Pediatric Intensive Care, Erasmus MC - Sophia Children’s Hospital, University Medical Center Rotterdam, Rotterdam, The Netherlands

**Keywords:** COVID-19, Long COVID, Healthcare services, Implementation, Aftercare, Patient satisfaction, Unmet needs

## Abstract

**Background:**

Patient experiences with COVID-19 aftercare remain largely unknown. We evaluated COVID-19 aftercare from a patient perspective one year after hospitalization, assessing satisfaction and its associated factors, and unmet needs.

**Methods:**

The Satisfaction with COVID-19 Aftercare Questionnaire (SCAQ) was developed as part of a multicenter prospective cohort study and administered one year after hospital discharge. The SCAQ assesses (1) patient satisfaction, comprising information provision, rehabilitation, follow-up by hospitals and general practitioners (GPs), the most important aftercare topics, and overall satisfaction, and (2) unmet needs.

**Results:**

487/561 (87%) COVID-19 patients completed the SCAQ, all had been discharged from the hospital between March 2020 and May 2021. Among responders, the median age of patients was 60 (IQR 54–67) years, 338 (69%) were male, and the median length of stay in the hospital was 13 (6–27) days. Patients were least satisfied with information on who could be contacted with questions when health problems arise (59% satisfied or very satisfied). Many patients (75%) received rehabilitation, most frequently community-based (70%). Across the different community-based therapies, ≥ 60% of patients were satisfied with shared-decision making and ≥ 70% with the received therapy; a majority (≥ 79%) indicated a preference for receiving the same therapy again if needed. Regarding follow-up by hospitals, 86% of patients received this follow-up, most frequently visiting a pulmonologist (96%), being generally satisfied with the received aftercare. Aftercare from GPs was received by 39% of patients, with 88% being satisfied with the GP’s availability and 79% with referral to appropriate aftercare providers. Patients (> 50%) considered information-related items most important in aftercare. Overall, patients rated their satisfaction with aftercare 8/10 (7–9) points. Those who received medical rehabilitation (versus no rehabilitation, adjusted beta 0.61 [95%CI 0.11 to 1.11], p = 0.02) or aftercare by a hospital medical specialist (1.1 [0.46 to 1.64], p < 0.001) or GP (0.39 [0.053 to 0.72], p = 0.023) reported significantly higher satisfaction than those without such aftercare. Unmet needs were reported by 35% of patients, with lack of information (20%) and lack of additional aftercare and/or involvement of their GP (19%) being the most frequently reported.

**Conclusion:**

Despite the forced quick development of COVID-19 aftercare, patients were generally satisfied. Follow-up by healthcare professionals and information provision is important to meet patients’ aftercare needs.

**Supplementary Information:**

The online version contains supplementary material available at 10.1186/s12931-024-02748-8.

## Highlights

- Our findings hold potential implications for enhancing aftercare after hospitalization for COVID-19 and for future pandemics.
- COVID-19 patients receiving aftercare from hospital medical specialists or general practitioners generally expressed higher satisfaction with COVID-19 aftercare than those who did not. Facilitating this aftercare is recommended to meet the patient’s aftercare needs.
- COVID-19 patients experienced unmet information needs after hospitalization, while they considered information most important in aftercare. Early implementation and promotion of a central online information point, providing up-to-date knowledge (e.g., on the disease, procedures, and recovery), can address the patients’ information needs.

**Supplementary Information:**

The online version contains supplementary material available at 10.1186/s12931-024-02748-8.

## Introduction

The COVID-19 pandemic, caused by SARS-CoV-2, resulted in a challenge for governments and healthcare systems worldwide to provide optimal long-term health care. Millions of people have been infected with SARS-CoV-2, ranging from asymptomatic infection to a life-threatening syndrome [1]. The pandemic forced the quick development of care pathways without adequate knowledge of the patients’ long-term care needs.

As we now know, many patients hospitalized for COVID-19 suffer from long-term health effects, comprising physical, cognitive, and mental problems, [1–5] known as long COVID or Post-COVID-19 Condition. [6, 7] Previous studies reported that 45%-90% of the patients hospitalized for COVID-19 experience at least one persistent symptom one year after discharge, underlining the importance of follow-up and tailored aftercare [2, 8–10]. The large demand for COVID-19 aftercare has required healthcare providers to rearrange their existing services, implementing programs that match the most appropriate level of care for these patients. Post-COVID-19 management has resulted in increased pressure on healthcare services along with higher healthcare costs [11–13].

Little is known about patients’ experiences with aftercare after hospitalization for COVID-19. Understanding the patients’ satisfaction with COVID-19 aftercare and unmet needs may help identify potential areas for improvement and may ultimately also improve healthcare services in future pandemics. This study aimed to explore 1) patients’ satisfaction with COVID-19 aftercare and its associated factors and 2) unmet needs in patients previously hospitalized for COVID-19. We used the COVID-19 Aftercare Questionnaire (SCAQ), which was specifically designed for this study.

## Methods

### Study design and participants

We performed a cross-sectional study among patients who had been hospitalized for COVID-19. The study was performed as part of an ongoing two-year prospective multicenter cohort study: COVID-19 Follow-up care paths and Long-term Outcomes within the Dutch health care system (CO-FLOW), conducted in the Rotterdam–Rijnmond–Delft region of the Netherlands. CO-FLOW participants were patients who had been hospitalized for COVID-19 in this region (1 academic hospital and 6 regional hospitals), aged 18 years or older, and with sufficient knowledge of the Dutch or English language. Patients were included between July 2020 and Oct 2021. CO-FLOW visits were performed at 3, 6, 12, and 24 months after hospital discharge and included physical and cognitive tests and an online survey. More information about the CO-FLOW study design can be found elsewhere [14]. The Medical Ethics Committee of Erasmus MC (MEC-2020-0487) approved the CO-FLOW study. All participants provided written informed consent before the start of study measurements. The study is registered on the World Health Organization International Clinical Trial Registry Platform (NL8710). Data were collected and stored using the Castor Electronic Data Capture system (Castor EDC, Amsterdam, The Netherlands).

### Study procedures

The SCAQ is an online questionnaire assessed as part of the one-year CO-FLOW study assessments. The questionnaire is only available in Dutch and therefore restricted to participants with sufficient knowledge of the Dutch language. Participants received the SCAQ by email approximately 3–4 weeks before the one-year visit and were invited to complete the questionnaire before the visit; an automatic reminder was sent after seven days. For participants who did not complete the online questionnaire by the time of the visit, the SCAQ was assessed by one of the research team members during the on-site study visit. This was also done for participants who previously received questionnaires per post.

### Routine care pathways

Following hospitalization for COVID-19, patients unable to be discharged home and requiring inpatient multidisciplinary rehabilitation were referred to a medical rehabilitation center (Med-rehab) or a skilled nursing facility (SNF-rehab) [15]. Patients who were sufficiently independent at discharge were discharged home with or without the support of community-based rehabilitation (Com-rehab), often mono-disciplinary treatment, or referred to outpatient Med-rehab for multidisciplinary rehabilitation. Following Med- or SNF-rehab, patients may have continued rehabilitation in the community.

In the region where this study is conducted, it is standard practice that the discharging hospital offers outpatient follow-up to COVID-19 patients. Some hospitals have designed specific, sometimes multidisciplinary, outpatient clinics for COVID-19 patients, while others embedded the follow-up in regular outpatient clinics. The follow-up program is hospital-specific; the timing of the first follow-up visit varies between 1 and 3 months post-discharge. Pulmonary function tests (spirometry and diffusion capacity), laboratory tests, and radiology (chest radiography and chest CT scans) are generally performed as part of the follow-up. A consultation, face-to-face or a telephone/video consultation due to COVID-19 restrictions, with a hospital medical specialist—most frequently a pulmonologist—is usually scheduled within one week thereafter. Patients with no or limited residual pulmonary abnormalities are discharged from further follow-up in the hospital. Apart from aftercare from hospital medical specialists, general practitioners (GPs) play a pivotal role in coordinating COVID-19 aftercare as many individuals typically reach out to their GP as their first contact when discussing health problems.

### Satisfaction with COVID-19 Aftercare Questionnaire (SCAQ)

We developed the SCAQ instrument in co-creation with a subgroup of the CO-FLOW study participants and an implementation specialist. The instrument assesses patient satisfaction and unmet needs regarding COVID-19 aftercare following hospitalization for COVID-19. We performed four online focus group interviews, each consisting of 4–5 participants, to conduct semi-structured interviews on predefined themes related to COVID-19 aftercare, e.g., information provision, rehabilitation, and post-discharge follow-up by hospital and GP. Participants were free to deviate from the predefined themes and interact with each other. The focus groups were audio recorded and transcribed. We included the predefined themes to develop a draft version of the SCAQ and added the additional themes as indicated by the participants during the focus groups. We conducted a pilot test with 9 participants to assess the questions’ clarity, comprehensibility, and feasibility. Textual modifications were made to the SCAQ if necessary.

The final version of the SCAQ assesses (1) patient satisfaction, comprising satisfaction with information provision, rehabilitation, and post-discharge follow-up by hospital and GP, as well as the most important aftercare topics and overall satisfaction, and (2) unmet needs. Patients scored their satisfaction regarding information provision, rehabilitation, and post-discharge follow-up by hospital and GP on a 5-point Likert scale: very dissatisfied, dissatisfied, not satisfied and not dissatisfied, satisfied, or very satisfied. Patients scored their overall satisfaction with COVID-19 aftercare on a 10-point numeric scale. Besides closed questions, patients could enter areas for improvement and other feedback in open-text fields. The number of SCAQ items varied across responders, depending on their specific aftercare received. The time to complete the questionnaire was approximately 10 min. The SCAQ items are presented in more detail in the Additional file 1: Methods.

#### Information provision

Satisfaction with information provision at hospital discharge or thereafter was assessed in 3 items (e.g., 'How satisfied are you with information about the recovery period?').

#### Rehabilitation

Satisfaction with rehabilitation was assessed for: A, shared decision-making ('How satisfied are you with discussing your treatment plan with your physician?'); B, the treatment ('How satisfied are you with the treatment you received?'); and C, whether patients would prefer to receive the same type of treatment again if they found themselves in similar circumstances.

For Med- and SNF-rehab, comprising multidisciplinary treatment, satisfaction regarding items A–C was rated for the overall rehabilitation program that is guided by an interdisciplinary team under supervision of a rehabilitation physician or geriatrician. Additionally, items A and B were also rated separately for each therapy during Med-rehab (in- and outpatient) and SNF-rehab (inpatient). For Com-rehab, satisfaction regarding items A-C was rated for each therapy that the patient received (e.g., physical and occupational therapy).

#### Follow-up by the hospital and GP

Patients were asked whether they had received follow-up by the hospital and, if so, which medical specialist (e.g., pulmonologist, cardiologist, or internist) they had visited. Satisfaction with aftercare from the hospital was assessed in 7 items (e.g., 'How satisfied are you with the timing of the first follow-up visit'). Patients who had not received follow-up by the hospital were asked whether they had received an invitation for a follow-up visit and about their willingness to participate in such follow-up.

Satisfaction with aftercare from the GP was assessed for the GP’s availability (e.g., for asking questions) and referral to appropriate aftercare providers.

#### Most important aftercare topics

Patients scored, with a maximum of 5, the most important aftercare topics (e.g., information about the recovery period and the possibility of getting in touch with peers) from a list of 22 items (Additional file 1: Methods).

#### Overall satisfaction with COVID-19 aftercare

Patients rated their overall satisfaction with COVID-19 aftercare on a numeric 10-point scale ranging from ‘very dissatisfied’ (1) to ‘very satisfied’ (10).

#### Unmet needs

Potential unmet needs were assessed in information provision, shared decision-making, additional aftercare and/or involvement of the GP, and practical matters (e.g., accessibility of healthcare provider). If one or more of these unmet needs were reported, more specific options (6 to 8 items) followed to further characterize the unmet need (Additional file 1: Methods).

### Demographics and clinical characteristics

Demographics and clinical characteristics were collected for descriptive reasons and to assess whether these characteristics were associated with overall satisfaction. We collected patient demographics (age, sex, body mass index [BMI], migration background, education level, living situation, and employment status) and clinical characteristics (comorbidities, timing of hospitalization [COVID-19 wave], oxygen therapy, invasive mechanical ventilation, COVID-19-directed treatment, intensive care unit [ICU] admission, ICU length of stay, and hospital length of stay) at hospital admission from electronic patient records in the participating hospitals and with complementary questionnaires. We classified patients as being hospitalized during the first COVID-19 wave (February to July 2020), the second wave (July 2020 to February 2021), and the third wave (February to June 2021).

### Statistical analysis

Continuous variables were not normally distributed and presented as medians with interquartile ranges (IQR); normal distribution of the data was checked with the Shapiro–Wilk test. Categorical variables are presented as numbers with percentages. Some data are presented as both median (IQR) and mean (standard deviation [SD]) to allow for a comprehensive representation of the data distribution. To assess differences in demographics and clinical characteristics between responders and non-responders (patients who did not complete the SCAQ or those lost to follow-up), we used the Mann–Whitney U test for continuous variables and a chi-square test for categorical variables. Regarding the most important aftercare topics, patients who selected more than 5 items were excluded from this specific analysis. We performed additional analyses to gain in-depth information on patient satisfaction. For follow-up by the hospital, we assessed the association between groups of satisfaction levels (very dissatisfied or dissatisfied, not satisfied and not dissatisfied, satisfied, or very satisfied) regarding the timing of this follow-up and the number of days between discharge and follow-up using Spearman’s rank correlation coefficient. We calculated the timing of follow-up in the hospital as the number of days between hospital discharge and the first follow-up assessment in the hospital. For overall satisfaction, we conducted a multivariable generalized estimating equations (GEE) analysis to assess determinants for overall satisfaction with COVID-19 aftercare (numeric 10-point scale). For this analysis, age, sex (male vs. female), ethnicity (European vs. non-European), pre-COVID living situation (together with partner or parent vs. alone with or without children), pre-COVID employment status (employed vs. unemployed vs. retired), timing of hospitalization (first vs. second vs. third COVID-19 wave) and aftercare factors including rehabilitation (no rehabilitation vs. Com-rehab vs. Med-rehab vs. SNF-rehab), and follow-up by the hospital (yes vs. no), or by a GP (yes vs. no) were entered as fixed factors in the model. Quotes from open-text fields were not systematically analysed, but manually selected to further illustrate the level of satisfaction with aftercare. A p < 0.05 was considered statistically significant. The statistical analysis was performed using IBM SPSS Statistics version 28 (SPSS Inc., Chicago, IL, USA).

## Results

### Participants

**Fig. 1:**
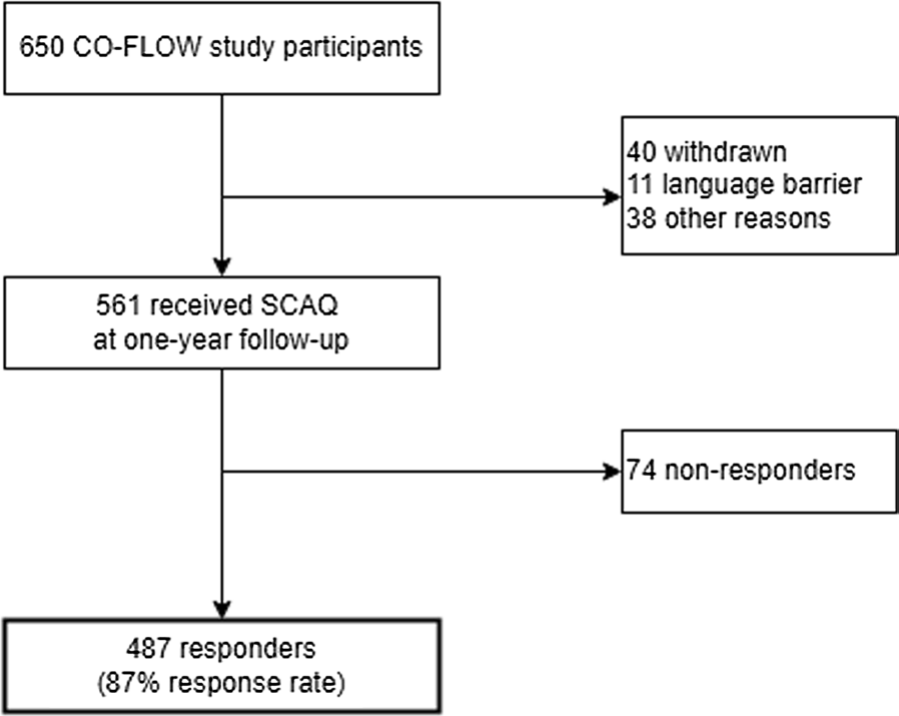
Flowchart of study participants. SCAQ: Satisfaction with COVID-19 Aftercare Questionnaire

A total of 561 out of 650 CO-FLOW study participants was invited to complete the SCAQ, of whom 487 (87%) responded; 89 patients were not invited due to reasons such as prior withdrawal (n = 40), language barrier (n = 11), or other reasons (n = 38) including inability to attend the one-year follow-up visit (Fig. 1). Table 1 presents the demographics and clinical characteristics at hospital admission of the 487 responders, all had been discharged from the hospital between March 2020 and May 2021. The median age of participants was 60 (IQR 54–67) years at admission, 338 (69%) were male, and 385 (79%) had a European background; 202 (42%) patients were treated in the ICU for COVID-19 and the median number of days in the hospital was 13 (6–27). Responders had more frequently a European background and middle to high education level and had less frequently diabetes in comparison to non-responders (n = 163) (Additional file 1: Table S1). Patients completed the SCAQ at a median follow-up time of 356 (339–399) days post-discharge. Among responders, 47/487 (10%) patients did not receive any type of aftercare, neither rehabilitation nor follow-up by the hospital or GP.

**Table 1:** Demographic and clinical characteristics of study participants at hospital admission

	Study participants (n = 487)
Demographic characteristics	
Age (years)	60 (54–67)
Male sex	338 (69)
BMI (kg/m^2^)	28 (26–32)
Ethnicity	
European	385 (79)
Dutch Caribbean	59 (12)
Asian	19 (4)
Turkish	13 (3)
(North) African	10 (2)
Education level	
Low	152 (32)
Middle	179 (37)
High	151 (31)
Living situation	
Together, with partner or parent	396 (81)
Alone, with or without children	91 (19)
Employed	294 (61)
Clinical characteristics	
Comorbidities	
≥ 1 comorbidity	399 (82)
Obesity (BMI ≥ 30 kg/m^2^)	194 (40)
Diabetes	82 (17)
Cardiovascular disease or hypertension	182 (37)
Pulmonary disease	121 (25)
Renal disease	44 (9)
Gastrointestinal disease	24 (5)
Neurological disease	51 (10)
Malignancy	57 (12)
Autoimmune or inflammatory disease	57 (12)
Mental disorder	22 (5)
Oxygen therapy	472 (97)
Treatment for COVID-19	
(Hydroxy)chloroquine	13 (3)
Antivirals	71 (15)
Steroids	353 (72)
Anti-inflammatory	57 (12)
Convalescent plasma	8 (2)
ICU admission	202 (41)
Invasive mechanical ventilation	173 (36)
LOS ICU (days)	16 (9–31)
LOS hospital (days)	13 (6–27)
COVID-19 wave	
First	129 (26)
Second	252 (52)
Third	106 (22)

Data are presented as median (interquartile range) or number (%). Variables with missing data are BMI (n = 47), ethnicity (n = 1), education level (n = 5), employed (n = 4), treatment for COVID-19 (n = 8), LOS ICU (n = 2), and LOS hospital (n = 1)

*BMI* body mass index, *COVID-19* corona virus disease 2019, *ICU* intensive care unit, *LOS* length of stay

### SCAQ

#### Information provision

**Fig. 2:**
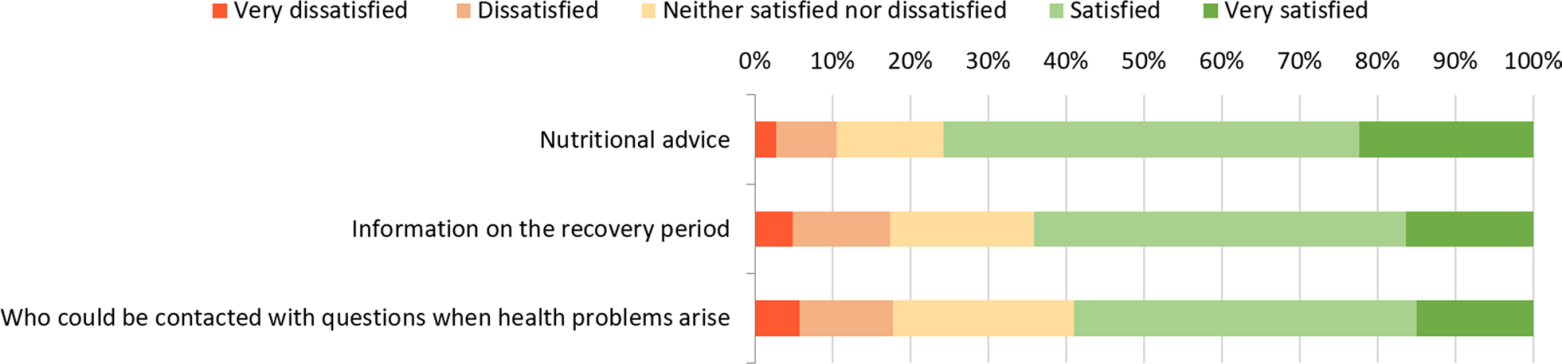
Patients’ satisfaction with information provision after hospitalization for COVID-19. Satisfaction with each item was assessed only for patients to whom it applies (self-indicated). Items were applicable to 362 (74%) of 487 patients on nutritional advice, 432 (89%) on information on the recovery period, and 439 (90%) on information on who could be contacted with questions when health problems arise

Most patients were satisfied with the information provided at hospital discharge or thereafter (Fig. 2). The lowest level of satisfaction (59% satisfied or very satisfied) was found for information on who could be contacted with questions when health problems arise.

#### Rehabilitation

Across the three rehabilitation settings Med-, SNF-, and Com-rehab, 342 (70%) patients participated in physical therapy, 124 (26%) in occupational therapy, 107 (22%) in psychological therapy, 71 (15%) in speech and language therapy, 124 (26%) in nutritional therapy, and 100 (21%) in vocational therapy; 123 (25%) patients did not receive rehabilitation. Table 2 presents the patients’ satisfaction with Med-, SNF-, and Com-rehab. Seventy-two (15%) patients received Med-rehab, of whom 89% reported a preference for receiving Med-rehab again if they found themselves in similar circumstances. Out of the 48 (10%) patients in SNF-rehab, 83% reported a preference for receiving SNF-rehab again if they found themselves in similar circumstances. The majority of patients (343/486, 71%) received Com-rehab after hospitalization for COVID-19, most frequently physical therapy (92%); a majority (95%) reported a preference for receiving physical therapy again if they found themselves in similar circumstances. As for vocational therapy, multiple patients reported in the open-text field that they had not (fully) resumed work one year after hospital discharge. These patients emphasized the importance of sharing knowledge about the health effects of COVID-19 with their employer. One patient reported: *‘The responsibility lies too much in the hands of the patient. There is little room for shared decision-making. Also, there is little time to discuss progress. There seems to be limited knowledge about COVID-19?’.*

**Table 2:** Frequency of patients that received rehabilitation after hospitalization for COVID-19

	n (%)^a^	(Very) Satisfied with shared decision-making^b^ n (%)	(Very) Satisfied with treatment^b^ n (%)	Preference for receiving the same treatment again (yes)^c^ n (%)
Med-rehab (n = 72)				
Rehabilitation physician	72 (100)	58 (82)	63 (89)	63 (89)
Physical therapy	71 (99)	62 (87)	64 (90)	
Occupational therapy	64 (89)	57 (89)	55 (86)	
Psychological therapy	50 (69)	45 (90)	44 (88)	
Speech and language therapy	31 (43)	27 (87)	28 (90)	
Nutritional therapy	44 (61)	35 (80)	35 (80)	
Other^*^	4 (6)			
SNF-rehab (n = 48)				
Elderly care physician	48 (100)	29 (60)	42 (88)	40 (83)
Physical therapy	47 (98)	37 (79)	41 (87)	
Occupational therapy	30 (63)	26 (87)	26 (87)	
Psychological therapy	19 (40)	14 (74)	17 (89)	
Speech and language therapy	19 (40)	13 (68)	15 (79)	
Nutritional therapy	30 (63)	27 (93)	28 (97)	
Other*	2 (4)			
Com-rehab^d^ (n = 343)				
Physical therapy	316 (92)	277 (88)	276 (87)	299 (95)
Occupational therapy	46 (13)	36 (78)	35 (76)	37 (80)
Psychological therapy	52 (15)	41 (79)	43 (83)	48 (92)
Speech and language therapy	32 (9)	30 (94)	31 (97)	29 (91)
Nutritional therapy	68 (20)	55 (81)	56 (82)	56 (82)
Vocational therapy	100 (29)	76 (76)	70 (70)	79 (79)
Other	3 (1)			

Data were obtained from 486 patients and are presented as n (%), where the percentage indicates the proportion of patients with this therapy among those who participate in this rehabilitation setting. Satisfaction was scaled on a 5-point Likert scale ranging from very satisfied, satisfied, not satisfied and not dissatisfied, dissatisfied, or very dissatisfied. The table presents the frequency of very satisfied or satisfied patients

*Med-rehab* in- or outpatient medical rehabilitation, *SNF-rehab* inpatient rehabilitation in a skilled nursing facility, *Com-rehab* community-based rehabilitation

^a^Frequency is calculated based on the number of patients that participated in that specific type of rehabilitation

^b^Frequency based on the number of patients that participated in that specific type of treatment

^c^Med- and SNF-rehab comprise multidisciplinary treatment that is guided by an interdisciplinary team, patients who received Med- or SNF-rehab were therefore asked whether they would like to receive Med- or SNF-rehab again if they found themselves in similar circumstances. Com-rehab often comprise monodisciplinary treatment and patients were therefore asked to indicate for each type of treatment whether they would like to receive the same therapy again if they found themselves in similar circumstances

^d^Com-rehab also comprises patients that may have participated in Med- or SNF-rehab. These patients scored satisfaction with Med- or SNF-rehab and Com-rehab separately

*Most frequently a social worker

#### Follow-up by the hospital and GP

**Fig. 3:**
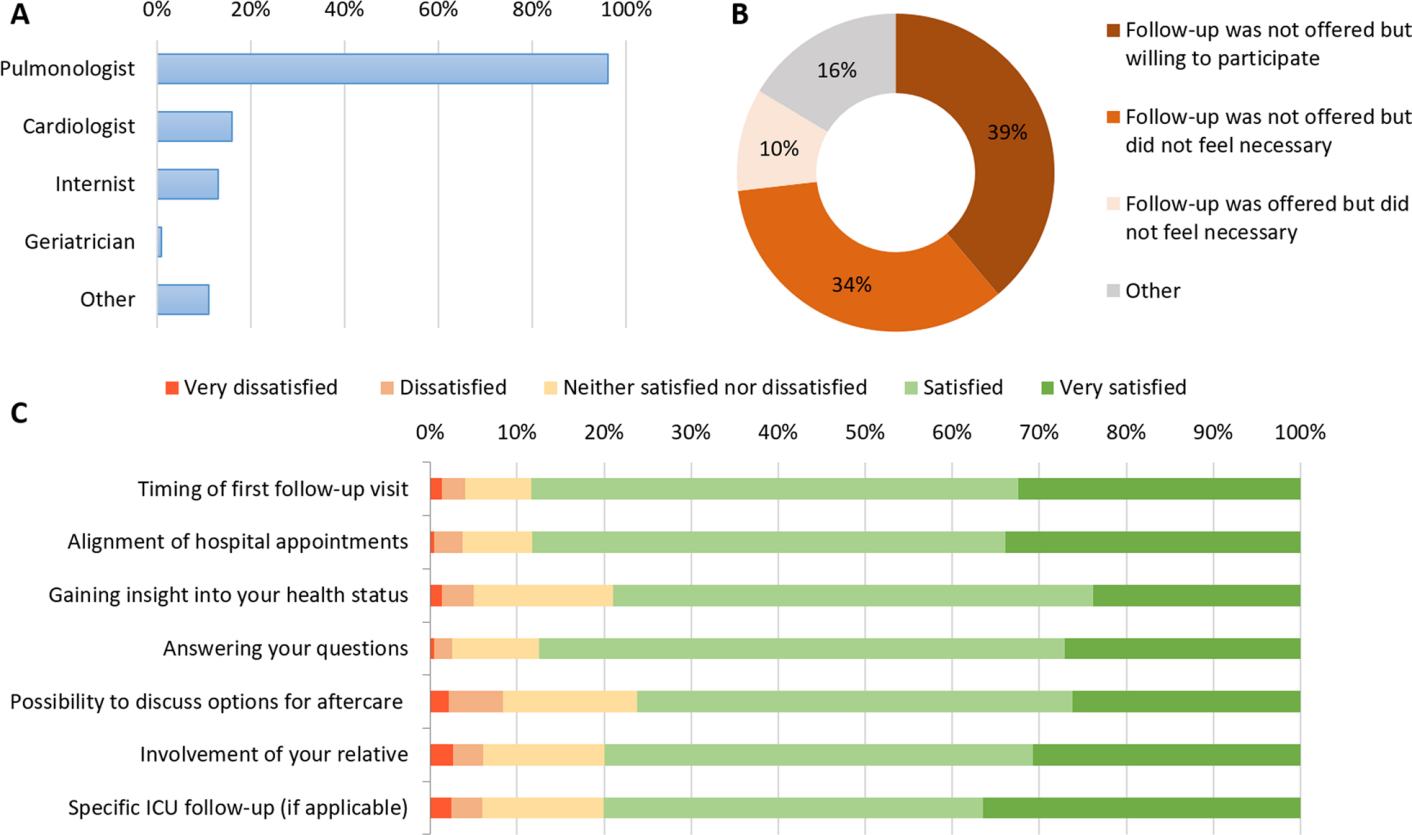
Follow-up by the hospital with **A**: the proportion of 420 COVID-19 patients that visited medical specialists, **B**: reasons for not participating in this follow-up, and **C**: patients’ satisfaction with aftercare provided by their medical specialist. **A**: 420 (86%) patients received follow-up by the hospital, of whom 292 (70%) patients visited one specialist, 77 (18%) two specialists, 33 (8%) three specialists, 14 (3%) four specialists, and 4 (1%) 5 specialists. **B**: Sixty-seven patients did not receive this follow-up; one example for ‘other reasons’ included ‘*follow-up *via* surveys only.*’ **C**: data are presented for 420 patients who participated in follow-up by the hospital and to whom it applies (self-indicated)

In total, 420/487 (86%) patients received follow-up by the hospital, the majority of whom (96%) visited a pulmonologist (Fig. 3A). In contrast, 67/487 (14%) patients did not undergo this follow-up; in 39% of these patients, follow-up was not initially offered but the patient was willing to participate (Fig. 3B). Regarding aftercare provided by the hospital medical specialist, the lowest level of satisfaction was found for the possibility of discussing options for aftercare with their medical specialist (76% satisfied or very satisfied, Fig. 3C). The median follow-up time was 51 (43–66) days (mean 60 ± 28.9 days) after hospital discharge. We found that increased satisfaction with the timing of the first follow-up visit significantly correlated with earlier follow-up in days (r = 0.15, p = 0.003) (Additional file 1: Table S2).

Regarding GPs, 191/486 (39%) patients received their aftercare, of whom 88% reported being satisfied or very satisfied with their availability and 79% with their referral to appropriate aftercare providers. Several patients expressed gratitude toward the GP in the open-text field; one patient reported: ‘*The GP called me after my discharge from the hospital. She referred me to physical therapy and providers that have experience in guiding COVID-19 patients. This option was briefly mentioned in the hospital, but the GP discussed this extensively and recommended this type of therapy. For this I am very grateful.’* However, a minority of patients was not satisfied with the services of the GP and pointed out that they would have liked their (closer) involvement after hospital discharge.

#### Most important aftercare topics

The most important aftercare topics were information-related, including information about potential problems after hospital discharge (51%), the recovery period (49%), and who could be contacted with questions (29%) (Additional file 1: Fig. S1). Moreover, patients considered the involvement of their GP after hospital discharge (28%) and gaining insight into one’s own health status and recovery by healthcare providers (24%) among the most important topics.

#### Overall satisfaction with COVID-19 aftercare

**Fig. 4:**
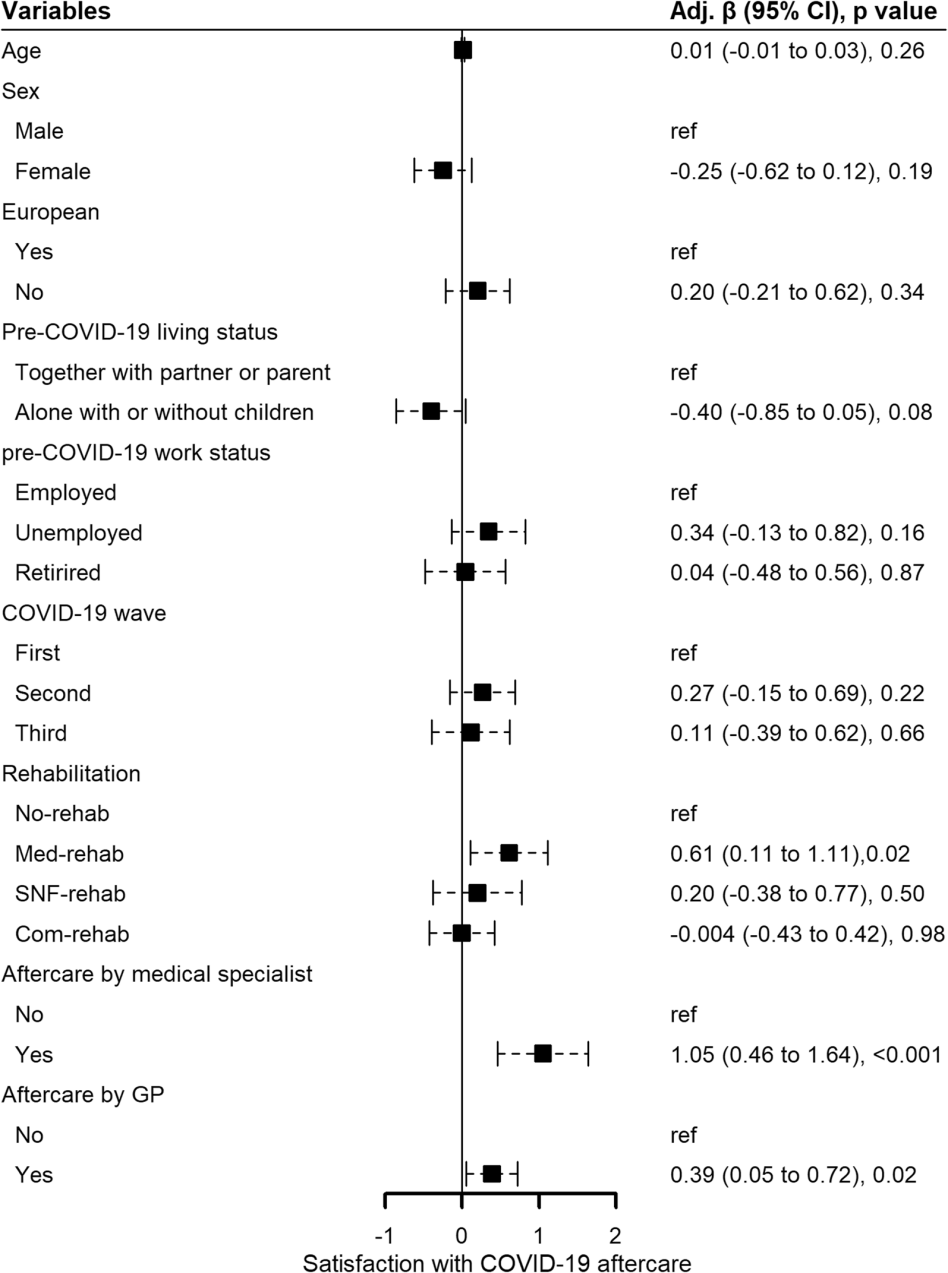
Forest plot showing predictors of the patients’ overall satisfaction with COVID-19 aftercare. The multivariable generalized estimating equations analysis included 481 patients. Satisfaction was assessed on a numeric scale from 0 to 10. For rehabilitation, patients were categorized according to the most specialized aftercare they had received after hospitalization for COVID-19, with Med- and SNF-rehab being the most specialized. Aftercare by a medical specialist indicates post-discharge follow-up provided by the hospital. *Adj. β* adjusted beta, *CI* confidence interval, *No-rehab* patients who did not receive rehabilitation, *Com-rehab* community-based rehabilitation, *Med-rehab* in- or out-patient medical rehabilitation, *SNF-rehab* inpatient rehabilitation in a skilled nursing facility, *GP* general practitioner

Patients rated their COVID-19 aftercare with a median of 8/10 (7–9) points (mean 7.3 ± 1.9 points). In the multivariable analysis, Med-rehab (vs. no rehabilitation mean difference 0.61 [95%CI 0.11 to 1.11], p = 0.02; vs. Com-rehab 0.61 [0.16 to 1.1], p = 0.008; vs. SNF-rehab 0.41 [− 0.20 to 1.02], p = 0.19) or follow-up by the hospital (1.1 [0.46 to 1.64], p < 0.001) or GP (0.39 [0.053 to 0.72], p = 0.023) predicted higher satisfaction with COVID-19 aftercare than those without this aftercare (Fig. 4).

#### Unmet needs

**Fig. 5:**
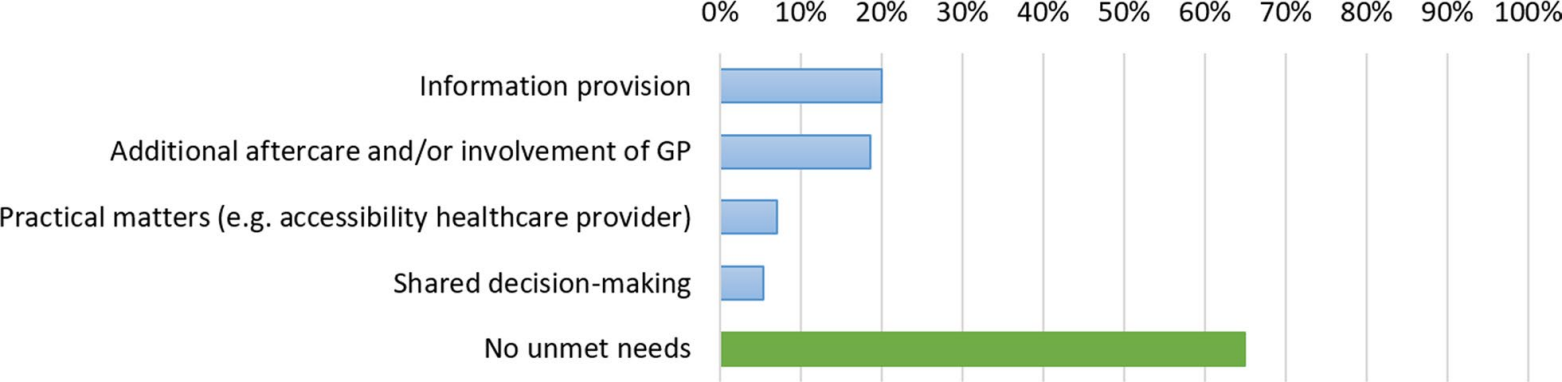
Unmet needs of patients recovering from COVID-19. Unmet needs are presented for 485 patients

Data on unmet needs were available for 485 patients, of whom 170 (35%) reported unmet needs following hospitalization for COVID-19 (Fig. 5). The most common unmet needs were information provision (20%) and additional aftercare and/or involvement of GP (19%). Specifically, 15% of all patients missed information about the potential problems after hospital discharge, 15% about the recovery period at home (e.g., how to optimize recovery), and 14% missed (close) involvement of the GP after hospital discharge.

## Discussion

This multicenter cohort study evaluated COVID-19 aftercare from a patient perspective one year after hospital discharge, assessing patient satisfaction and unmet needs. Overall, patients were generally satisfied with their COVID-19 aftercare, rating their overall satisfaction 8 out of 10 points. This while the aftercare was developed quickly and mainly based on expert opinion. Patients who received medical rehabilitation or aftercare from the hospital or GP generally expressed higher overall satisfaction with COVID-19 aftercare than those who did not receive such aftercare. We found that 35% of the COVID-19 patients experienced unmet needs after hospitalization, most frequently the lack of information and the lack of involvement of the GP. These unmet needs were also considered most important in aftercare by the patients, indicating areas for improvement.

It may not be surprising that patients who received more intensive aftercare had a more positive perception of their aftercare than those who did not receive such care. Regarding rehabilitation, patients with medical rehabilitation often had severe COVID-19, which had a significant physical and mental impact, requiring intensive and multidisciplinary rehabilitation to support recovery [3, 4]. Our findings indicate that this intensive and multidisciplinary type of rehabilitation is perceived as valuable by the most severely affected patients.

As for follow-up by hospitals, patients can gain insight into their health status, ask questions, and discuss options for aftercare with their medical specialist during this consultation. Our patients were generally satisfied with this aftercare. It is internationally recommended that hospitalized COVID-19 patients are routinely followed up at the discharging hospital [16–18]. Differences have been reported among European countries regarding the timing of the first follow-up visit by hospitals, varying between 1 to 6 months [19]. In our study, the patient’s first follow-up visit by the hospital was, on average, 60 days after discharge. Although we found a positive correlation between satisfaction with the timing of the first follow-up and earlier follow-up, the correlation was weak and, therefore, we cannot offer strong recommendations for the timing of follow-up by hospitals.

Regarding GPs, only 39% of COVID-19 patients received aftercare from their GP, with the majority being satisfied with the GP’s availability and referral to appropriate aftercare providers. Notably, the lack of involvement of a GP was one of the most frequently reported unmet needs as well as considered among the most important topics in aftercare by patients. GPs have a central role in coordinating COVID-19 aftercare, as patients usually contact their GP as their initial point of contact for health problems.

Our findings thus imply that a follow-up consultation with a healthcare professional is important to meet the COVID-19 patients’ aftercare needs. However, we did not explore whether only medical specialists or GPs should provide this aftercare or if other healthcare professionals, such as case managers, could also offer this aftercare. Noteworthy, this type of aftercare is likely valuable not only for COVID-19 patients but also for those with other conditions.

Our findings indicate unmet information needs, while information provision was considered the most important in aftercare. Studies in the Netherlands, Belgium, and the United Kingdom also showed that individuals post-COVID-19 seek information [20, 21]. Generally, meeting patients’ information needs has been linked to their satisfaction with care and quality of life [22]. Online health platforms and patient support groups have been established across countries to provide COVID-19-related information to the general public, particularly patients and their families. Furthermore, on social media like Facebook, communities were created where COVID-19 patients can connect, share experiences, and offer peer support. Nevertheless, our patients still reported unmet information needs. Notably, we enrolled patients since the onset of the COVID-19 pandemic, a time where aftercare pathways were still in the process of development. Utilizing online health platforms also faces challenges, particularly in reaching diverse populations. For example, older individuals, ethnic minorities, and those from socioeconomically disadvantaged backgrounds are generally less likely to access online health information [23–28]. Nevertheless, these online health platforms and communities serve as valuable sources of information on the developing knowledge of COVID-19, informing many patients, enhancing understanding and reassurance, facilitating self-management, and supporting recovery from COVID-19 [21, 29, 30]. We recommend the early implementation of a centralized online information point, a so-called live resource center, and increasing awareness of this online health platform during future pandemics.

We did not observe an association between patient characteristics such as age, sex, or migration background with patients’ satisfaction with COVID-19 aftercare. Noteworthy, our study may have had limitations in assessing the effect of migration background on patient satisfaction as we only included patients with sufficient knowledge of the Dutch language. Moreover, we classified migration backgrounds as European or non-European in the statistical analysis due to the small group sizes of ethnic minorities. A recent study among hospitalized COVID-19 patients in Amsterdam, the Netherlands, showed that ethnic groups, including African Surinamese, South Asian Surinamese, Moroccan, and Turkish origin patients, had a higher risk of long COVID than Dutch origin patients at 12 weeks post-discharge [31]. This suggests that ethnic minorities in the Netherlands may have potentially greater COVID-19 aftercare needs than the general population. Moreover, ethnic minorities may face challenges in accessing healthcare providers due to language barriers, which may influence their satisfaction with COVID-19 aftercare, which we possibly have missed in our study. Therefore, future research involving larger groups of patients with diverse migration backgrounds is warranted to better address their satisfaction with COVID-19 aftercare needs.

Besides language restrictions, our study is limited by the challenge of generalizing our findings from patients hospitalized for COVID-19 in the Netherlands to other countries. The variation in the course of SARS-CoV-2 variants, infection rates, population characteristics, and healthcare systems across countries resulted in heterogeneous follow-up programs and may hamper international comparisons. Furthermore, our study included patients hospitalized for COVID-19 during the first three COVID-19 waves in the Netherlands; those hospitalized afterward might have different aftercare experiences due to developing aftercare procedures. The median age of our study participants was 60 years; therefore, our findings may be less generalizable to younger age groups. Our study contains an overrepresentation of patients who had been admitted to the ICU compared to other cohort studies on hospitalized COVID-19 patients, [2, 32] resulting in a somewhat higher proportion of males in our sample. Our academic center served as a regional referral center for ICU patients, and many study participants were included from this center. However, our patients received various COVID-19 aftercare to support their recovery, providing insights into experiences across different rehabilitation settings. The SCAQ was developed specifically for this explorative study to gain insight into patients’ experiences with aftercare following hospitalization for COVID-19 in the Netherlands, as part of the one-year CO-FLOW study visit. Due to time constraints, assessing the validity and reliability of the questionnaire was not feasible. Given the persistent health effects of COVID-19 beyond one year [2], signifying a potential need for prolonged aftercare, future studies are warranted to evaluate patients’ healthcare utilization and satisfaction with aftercare in the longer term. Nonetheless, our study’s conclusive findings emphasize the importance of delivering follow-up care and information provision, which hold significance to the broader public as these align with international guidelines [30].

Strengths of the study include its multicentre design and high response rate (87%). The study provides insight into the patients’ evaluation of various aspects of COVID-19 aftercare and identifies unmet needs that could facilitate the improvement of care pathways during future pandemics.

In conclusion, we evaluated aftercare following hospitalization for COVID-19 from a patient perspective, leading to two main findings. First, the results emphasized the significance of follow-up after hospitalization to meet COVID-19 patients’ aftercare needs. Facilitating a follow-up consultation with a healthcare professional for hospitalized patients during future pandemics is recommended to provide recognition, understanding, and appropriate aftercare to patients. Second, patients hospitalized for COVID-19 experienced unmet information needs. Early implementation of online health platforms across countries, serving as a central information point, could be vital to meeting patients’ information needs during future pandemics.

### Supplementary Information

**Additional file 1: Table S1 **Comparison of demographics and clinical characteristics between responders and non-responders. **Table S2 **Satisfaction with the timing of the first follow-up visit by the hospital and follow-up in days. **Figure S1 **Most important aftercare topics.

## Data Availability

The datasets used and/or analysed during the current study are available from the corresponding author on reasonable request.

## Abbreviations

BMI: Body mass index
Com-rehab: Community-based rehabilitation
COVID-19: Coronavirus disease 2019
GP: General practitioner
ICU: Intensive care unit
IQR: Interquartile range
Med-rehab: Rehabilitation in medical rehabilitation center
SNF-rehab: Rehabilitation in a skilled nursing facility
SARS-CoV-2: Severe Acute Respiratory Syndrome Coronavirus 2
SD: Standard deviation
