# Supplementary material for: Patients’ evaluation of aftercare following hospitalization for COVID-19: satisfaction and unmet needs
Source: Respir Res. 2024 Mar 29;25:145. doi: 10.1186/s12931-024-02748-8 (PMC10981299; doi:10.1186/s12931-024-02748-8)
Supplement: Supplementary file 1 — Additional file 1: Table S1 Comparison of demographics and clinical characteristics between responders and non-responders. Table S2 Satisfaction with the timing of the first follow-up visit by the hospital and follow-up in days. Figure S1 Most important aftercare topics. [file 12931_2024_2748_MOESM1_ESM.docx]

**Supplementary material**

**Patients’ Evaluation of Aftercare Following Hospitalization for COVID-19: Patient satisfaction and Unmet Needs**

Julia C. Berentschot,^1^ Willemijn A. de Ridder,^2,3,4,5^ L. Martine Bek,^2^ Majanka H. Heijenbrok-Kal,^2,6^ G. J. Braunstahl,^7^ S.C. Remerie^3^, Y. Stuip,^8^ Gerard M. Ribbers,^2,6^ Joachim G.J.V. Aerts,^1^ Erwin Ista,^9,10^ Merel E. Hellemons,^1^ and Rita J.G. van den Berg-Emons,^2^ on behalf of the CO-FLOW Collaboration Group

^1^ Department of Respiratory Medicine, Erasmus MC, University Medical Center Rotterdam, Rotterdam, the Netherlands

^2^ Department of Rehabilitation Medicine, Erasmus MC, University Medical Center Rotterdam, Rotterdam, the Netherlands

^3^ Department of Plastic, Reconstructive and Hand Surgery, Erasmus MC, University Medical Center Rotterdam, Rotterdam, The Netherlands;

^4^ Hand and Wrist Center, Xpert Clinics, Eindhoven, The Netherlands;

^5^ Center for Hand Therapy, Xpert Handtherapie, Eindhoven, The Netherlands

^6^ Rijndam Rehabilitation, Rotterdam, the Netherlands

^7^ Department of Respiratory Medicine, Franciscus Gasthuis, Rotterdam, The Netherlands.

^8^ General practitioner, Zorghoek Bergschenhoek, Bergschenhoek, The Netherlands.

^9^ Department of Internal Medicine, Nursing Science, Erasmus MC, University Medical Center Rotterdam, Rotterdam, The Netherlands.

^10^ Departments of Neonatal and Pediatric Intensive Care, division of Pediatric Intensive Care, Erasmus MC - Sophia Children's Hospital, University Medical Center Rotterdam, Rotterdam, The Netherlands

| **Table of contents** | **Page** |
| --- | --- |
| Supplementary Methods | 1 |
| Supplementary Results |  |
| **Table S1** Comparison of demographics and clinical characteristics between responders  and non-responders | 6-7 |
| **Table S2** Satisfaction with the timing of the first follow-up visit by the hospital and  follow-up in days | 8 |
| **Figure S1** Most important aftercare topics | 9 |

**Supplementary methods**

The Satisfaction with COVID-19 Aftercare Questionnaire (SCAQ) is a newly developed questionnaire, specifically for the CO-FLOW study. We developed the SCAQ instrument in co-creation with a subgroup of the CO-FLOW study participants and an implementation specialist. The instrument assesses patient satisfaction and unmet needs with COVID-19 aftercare following hospitalization for COVID-19. A detailed description of the items included in the SCAQ is provided below.

**Information provision**

Satisfaction with information provision at hospital discharge or thereafter was assessed in 3 items. Patient satisfaction was assessed on a 5-point Likert scale with the response options very dissatisfied, dissatisfied, not satisfied and not dissatisfied, satisfied, and very satisfied. The answer option ‘not applicable’ was added for each item.

*How satisfied are you with …*

- Nutrition advice for at home?
- Information about the recovery period?
- Advice on who could be contacted with questions when health problems arise?

*If you have any remarks, please indicate:* [open text field]

**Rehabilitation**

This was assessed across three different rehabilitation settings: in- and/or outpatient medical rehabilitation (Med-rehab), rehabilitation in a skilled nursing facility (SNF-rehab), and community-based rehabilitation (Com-rehab). For Med- and SNF-rehab, the same questions were applied. Patient satisfaction was assessed on a 5-point Likert scale with the response options very dissatisfied, dissatisfied, not satisfied and not dissatisfied, satisfied, and very satisfied.

Med- and SNF-rehab

*Regarding the rehabilitation program overall, …*

- How satisfied are you with discussing your treatment plan with your physician?
- How satisfied are you with the treatment you received?
- Would you like to receive the same type of rehabilitation again if found in similar circumstances? (answer options: yes or no)

*Which type of therapy did you receive?*

- Physical therapy
- Psychological therapy
- Occupational therapy
- Speech and language therapy
- Nutritional therapy
- Other, that is ..

For each therapy, the following items were assessed:

- How satisfied are you with discussing your treatment plan with your physician?
- How satisfied are you with the treatment you received?
- If you have any remarks (e.g point for improvement), please indicate: [open text field]

Com-rehab

*Which type of therapy did you receive?*

- Physical therapy
- Psychological therapy
- Occupational therapy
- Speech and language therapy
- Nutritional therapy
- Other, that is .. [open text field]

For each therapy, the following items were assessed:

- How satisfied are you with discussing your treatment plan with your therapist?
- How satisfied are you with the treatment you received?
- Would you like to receive this therapy again if found in similar circumstances? (answer options: yes or no)
- If you have any remarks (e.g point for improvement), please indicate: [open text field]

**Post-discharge follow-up**

This was evaluated for follow-up in the hospital and the general practitioner. Patient satisfaction was assessed on a 5-point Likert scale with the response options very dissatisfied, dissatisfied, not satisfied and not dissatisfied, satisfied, and very satisfied.

Aftercare by the hospital

*Did you participate in post-discharge follow-up in the hospital?* (answer options: yes or no)

If not, *What was the reason for not participating in post-discharge follow-up?*

- Follow-up was not offered, but I was willing to participate
- Follow-up was not offered, did not consider it necessary
- Follow-up offered, did not consider it necessary
- Other reasons

If yes, *which medical specialist(s) did you visited?*

- Pulmonologist
- Cardiologist
- Internist
- Geriatrician
- Other

*How satisfied are you with ..*

- The timing of your first follow-up visit?
- Alignment of hospital appointments?
- Gaining insight into your health status?
- Answering your questions?
- Ability to discuss options for aftercare?
- Involvement of your relative?
- Specific ICU follow-up (if applicable)?

*If you have any remarks, please indicate:* [open text field]

Aftercare by the general practitioner (GP)

*How satisfied are you with the GP’s..*

- Availability (e.g. to ask questions)?
- Referral to appropriate aftercare providers, if you felt the need?

*If you have any remarks, please indicate:* [open text field]

**Most important aftercare topics**

*Choose up to 5 topics that you value the most in aftercare*

- Information about the expected problems after hospital discharge
- Nutritional advice during recovery
- Information about the recovery period (e.g. how to optimize recovery)
- Information about who could be contacted with questions
- Information about aftercare health insurance reimbursement
- The possibility of getting in touch with peers
- Participation in the choice of discharge location after hospitalization
- Shared decision-making in a treatment plan
- Involvement of relatives in aftercare (e.g. in a conversation with your practitioner)
- Gaining insight into one’s own health status and recovery by healthcare providers
- Answering of questions
- Home care
- Direct involvement of GP after hospital discharge
- Aftercare offered by the rehabilitation center (if treated in rehabilitation center)
- Psychological and emotional aftercare
- Specific ICU aftercare (e.g. ICU follow-up visit, virtual reality or visiting the ICU)
- Adequate transportation from the hospital to the post-discharge location
- Request for medical devices at home
- Alignment of hospital appointments
- Accessibility and responsiveness of healthcare providers
- Accurate handover of information between involved healthcare professionals
- Other, that is .. [open text field]

**Overall rating of COVID-19 aftercare**

This item was assessed with a slide bar, representing a numeric rating scale from ‘very dissatisfied’ (1) at left to ‘very satisfied’ (10) at right, step size 1.

*What rating do you give your COVID-19 aftercare, on a scale from 1 (very dissatisfied) to 10 (very satisfied)? Move the bar to the appropriate grade.*

**Unmet needs**

*If you missed aftercare during your recovery from COVID-19, what did you miss?*

- Information provision
- Shared decision making
- Additional aftercare or involvement of the GP
- Practical matters (e.g. transport after discharge, accessibility of healthcare provider)
- No unmet needs

If one or more unmet needs were reported, the patient received more specific options to further characterize the unmet need.

*Specify unmet need in information provision*

- Information about the potential problems after hospital discharge
- Nutritional advice during recovery
- Information about the recovery period (e.g. how to optimize recovery)
- Information about who could be contacted with questions
- Information about aftercare health insurance reimbursement
- The possibility of getting in touch with fellow COVID-19 survivors
- Other, that is …. [open text field]

*Specify unmet need in shared decision-making*

- Shared decision-making in the choice of discharge location after hospitalization
- Shared decision-making in a treatment plan
- Involvement of relatives in aftercare (e.g. in a conversation with your practitioner)
- Gaining insight into one’s own health status and recovery by healthcare provider
- Answering of questions
- Other, that is … [open text field]

*Specify unmet need in additional aftercare or involvement of the GP*

- Direct involvement of GP after hospital discharge
- Aftercare offered by the rehabilitation center (if treated in rehabilitation center)
- Psychological and emotional aftercare
- Specific ICU aftercare (e.g. ICU follow-up visit, virtual reality or visiting the ICU)
- Other, that is …. [open text field]

*Specify unmet need in practical matters (e.g. transport after discharge, accessibility of healthcare provider)*

- Adequate transportation from the hospital to the post-discharge location
- Request for medical devices at home
- Alignment of hospital appointments
- Accessibility and availability of healthcare provider
- Accurate handover of information between involved healthcare professionals
- Other, that is …. [open text field]

**Supplementary results**

**Table S1** Comparison of demographics and clinical characteristics between responders and non-responders.

|  | **Responders**  **(n=487)** | **Non-responders**  **(n=163)** | **P value** |
| --- | --- | --- | --- |
| Age (years) | 60 (54-67) | 60 (49-69) | 0.31 |
| Sex, male | 338 (69) | 110 (67) | 0.65 |
| BMI (kg/m²) | 28 (26-32) | 29 (25-32) | 0.68 |
| Migration background |  |  | <0.001^*^ |
| European | 383 (79) | 66 (45) |  |
| (North) African | 10 (2) | 15 (10) |  |
| Dutch Caribbean | 59 (12) | 30 (21) |  |
| Asian | 19 (4) | 20 (14) |  |
| Turkish | 13 (3) | 15 (10) |  |
| Education |  |  | <0.001 |
| High | 151 (31) | 34 (24) |  |
| Middle | 179 (37) | 39 (27) |  |
| Low | 152 (32) | 70 (49) |  |
| Living situation |  |  | 0.052 |
| Together with partner or parent | 396 (81) | 121 (74) |  |
| Alone with or without children | 91 (19) | 42 (26) |  |
| Employed | 294 (61) | 78 (54) | 0.15 |
| *Clinical characteristics* |  |  |  |
| Comorbidities |  |  |  |
| ≥1 comorbidity | 399 (82) | 135 (86) | 0.26 |
| Obesity (BMI≥30) | 194 (40) | 71 (44) | 0.40 |
| Diabetes | 82 (17) | 48 (29) | <0.001 |
| Cardiovascular disease or  hypertension | 182 (37) | 74 (45) | 0.07 |
| Pulmonary disease | 121 (25) | 41 (25) | 0.94 |
| Renal disease | 44 (9) | 15 (9) | 0.95 |
| Gastrointestinal  disease | 24 (5) | 7 (4) | 0.74 |
| Neurological disease | 51 (11) | 17 (10) | 0.99 |
| Malignancy | 57 (12) | 12 (7) | 0.12 |
| Autoimmune or inflammatory disease | 57 (12) | 11 (7) | 0.07 |
| Mental disorder | 22 (5) | 10 (6) | 0.41 |
| Oxygen suppletion | 472 (97) | 155 (95) | 0.27 |
| IMV | 173 (36) | 62 (38) | 0.56 |
| ICU admission | 202 (41) | 71 (44) | 0.64 |
| LOS ICU (days) | 16 (9-31) | 17 (9-32) | 0.56 |
| LOS hospital (days) | 13 (6-27) | 10 (5-31) | 0.58 |
| Wave of COVID-19 |  |  | 0.17 |
| First | 129 (26) | 51 (31) |  |
| Second | 252 (52) | 87 (54) |  |
| Third | 106 (22) | 25 (15) |  |

Data are presented as median (interquartile range) or number (%). Demographics and clinical characteristics in COVID-19 patients at the time of hospital admission stratified for responders and non-responders (i.e., patients who did not complete the SCAQ or those lost to follow-up). P values were obtained using the Mann-Whitney U test for continuous variables and the chi-square test for categorical variables. SCAQ: Satisfaction with COVID-19 Aftercare Questionnaire.

^*^Group comparison was performed as European versus non-European

**Table S2** Satisfaction with the timing of the first follow-up visit by the hospital and follow-up in days.

|  | n (%) | Number of days between hospital discharge and  follow-up in the hospital | |
| --- | --- | --- | --- |
|  |  | Mean ± standard deviation | Median (interquartile range) |
| **Satisfaction with the timing of follow-up** |  |  |  |
| Very satisfied | 122 | 56.1 ± 24.9 | 47 (42-60) |
| Satisfied | 218 | 60.3 ± 28.9 | 52 (43-66) |
| Not satisfied not dissatisfied | 29 | 69.7 ± 35.8 | 57 (45-78) |
| Dissatisfied or very dissatisfied | 15 | 74.6 ± 37.9 | 63 (41-100) |
| Dissatisfied | 9 | 74.1 ± 26.2 | 58 (41-89) |
| Very dissatisfied | 6 | 90.3 ± 49.3 | 82 (43-137) |

Data are presented for 384 patients with COVID-19.

**Figure S1.** Most important aftercare topics.

**
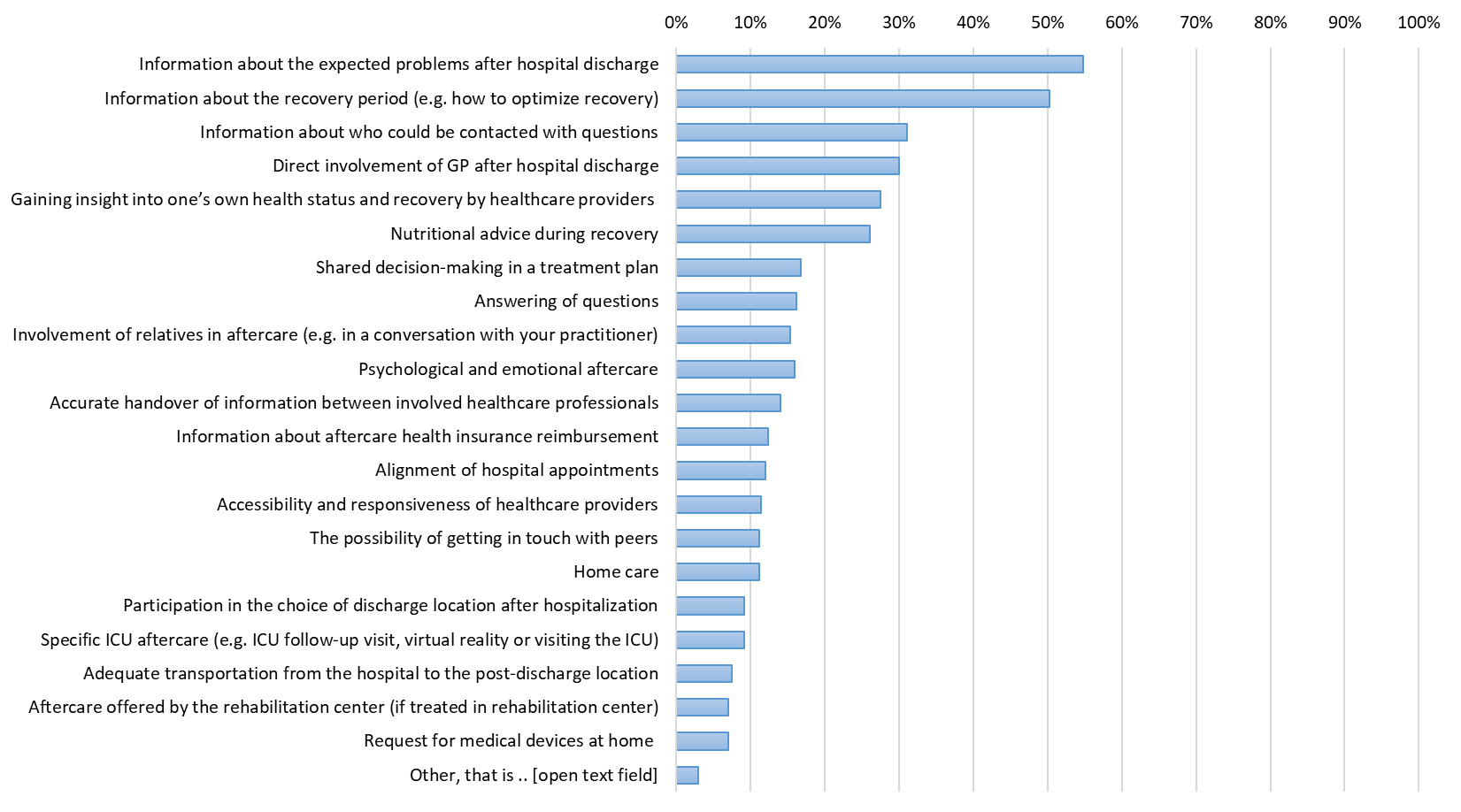
**

Data are presented as the frequency of 443 patients with COVID-19 who scored the most important aftercare topics with a maximum of 5 items.
